# Antimicrobial-Resistant Clonal Complex 11 *Neisseria meningitidis*–Associated Urethritis Cluster, Thailand

**DOI:** 10.3201/eid3110.250464

**Published:** 2025-10

**Authors:** John C. Cartee, Thitima Cherdtrakulkiat, Sandeep J. Joseph, Rossaphorn Kittiyaowamarn, Natnaree Girdthep, Pongsathorn Sangprasert, Porntip Paopang, Thidathip Wongsurawat, Piroon Jenjaroenpun, Perapon Nitayanon, Rebekah Frankson, Silvina Masciotra, Teodora Wi, Ismael Maatouk, Ellen N. Kersh, Andrey S. Borisov, Chanwit Tribuddharat

**Affiliations:** US Centers for Disease Control and Prevention, Atlanta, Georgia, USA (J.C. Cartee, T. Cherdtrakulkiat, S.J. Joseph, R. Frankson, S. Masciotra, E.N. Kersh, A.S. Borisov); Thailand Ministry of Public Health**­–**US Centers for Disease Control and Prevention Collaboration, Nonthaburi, Thailand (T. Cherdtrakulkiat, S. Masciotra, A.S. Borisov); Ministry of Public Health, Nonthaburi (R. Kittiyaowamarn, N. Girdthep, P. Sangprasert, P. Paopang); Mahidol University, Bangkok, Thailand (T. Wongsurawat, P. Jenjaroenpun, P. Nitayanon, C. Tribuddharat); World Health Organization, Geneva, Switzerland (T. Wi, I. Maatouk)

**Keywords:** *Neisseria meningitidis*, gonorrhea, bacteria, sexually transmitted infections, antimicrobial resistance, urethritis, meningococcal urethritis, AMR, Thailand

## Abstract

Sexually transmitted infections clinics in Bangkok, Thailand, reported increasing numbers of men with *Neisseria meningitidis*–associated urethritis during 2017–2023. Genomic analysis indicated global expansion of the nongroupable clonal complex 11 *N. meningitidis* urethritis clade. Continued global surveillance is needed to monitor the spread of antimicrobial-resistant *N. meningitidis* with urethral adaptability.

*Neisseria meningitidis* is known to cause severe invasive infections, such as meningitis, but asymptomatic *N. meningitidis* nasopharyngeal colonization occurs in ≈10% of the human population (*1*). *N. meningitidis*–associated urethritis cases also have been reported, including a cluster caused by nongroupable clonal complex 11 (CC11) *N. meningitidis* detected in the United States in 2015 (*2*,*3*). That US clade has since expanded globally, and *N. meningitidis* urethritis cases have been found in the United Kingdom, Japan, and Vietnam (*4*–*6*). Phylogenetic analysis revealed that the globally expanding clade has formed a distinct branch within the CC11 lineage, designated as the *N. meningitidis* urethritis clade (*Nm*UC) (*3*,*6*,*7*). Further genomic characterization of *Nm*UC revealed integration of multiple *Neisseria gonorrhoeae* genomic regions into genomes of *N. meningitidis* isolates via recombination, and those recombinations are thought to increase the ability of *N. meningitidis* to colonize the urethra (*3*,*6*,*7*).

Antimicrobial resistance (AMR) is not yet well established in *Nm*UC (*3*), but recombination between *N. meningitidis* and *N. gonorrhoeae* is a concern because AMR is prevalent in *N. gonorrhoeae* (*5*,*8*). In a 2019–2020 outbreak of *N. meningitidis–*associated urethritis in Vietnam, isolates from *Nm*UC had elevated MICs to ciprofloxacin (*5*). The isolates harbored common mutations found in *N. gonorrhoeae* that confer resistance to ciprofloxacin (*9*), which is concerning because ciprofloxacin is commonly used for prophylaxis against invasive meningococcal diseases (*8*). We analyzed isolates collected from a cluster of *N. meningitidis*–associated urethritis among men in Thailand to assess AMR and urethral adaptation.

## The Study

In 2015, Thailand began surveillance for *N. gonorrhoeae* urethritis as part of the World Health Organization Enhanced Gonococcal Antimicrobial Surveillance Programme (EGASP; https://www.who.int/initiatives/gonococcal-antimicrobial-surveillance-programme). Through EGASP surveillance, 31 urethritis-causing *N. meningitidis* isolates were collected from men in Thailand during 2017–2023 (*10*). All cases were successfully treated with 250 mg or 500 mg intramuscular ceftriaxone in accordance with national guidelines for male patients with urethral symptoms and gram-negative intracellular diplococci. The national reference laboratory (accredited according to ISO 15189:2012) confirmed *N. meningitidis* in isolates by using culture and biochemical characteristics of *Neisseria* spp. bacteria.

We selected 16 isolates for further investigation, ensuring representation from the earliest detected case to the most recent case (Appendix). We combined whole-genome long-read sequencing using a PromethION P2i with an R10.4.1 flow cell (both Oxford Nanopore Technologies, https://nanoporetech.com) and short-read sequencing using NovaSeq 6000 (Illumina Inc., https://www.illumina.com) and 150-bp paired-end reads for hybrid genome assembly according to standard protocols (*11*). We used PubMLST (https://pubmlst.org) to determine the specific gene alleles and genotype of the isolates (*12*). 

Multilocus sequence typing showed that 15 of 16 isolates belonged to CC11 and 1 to sequence type 35 clonal complex (CC35). Within the CC11 isolates, 3 isolates (NM13, NM14, and NM15) collected during April–June 2022 from men who had sex with women were separated by an average of 13 pairwise single-nucleotide polymorphisms (SNPs), representing a potential transmission cluster or locally circulating strain.

**Figure 1:**
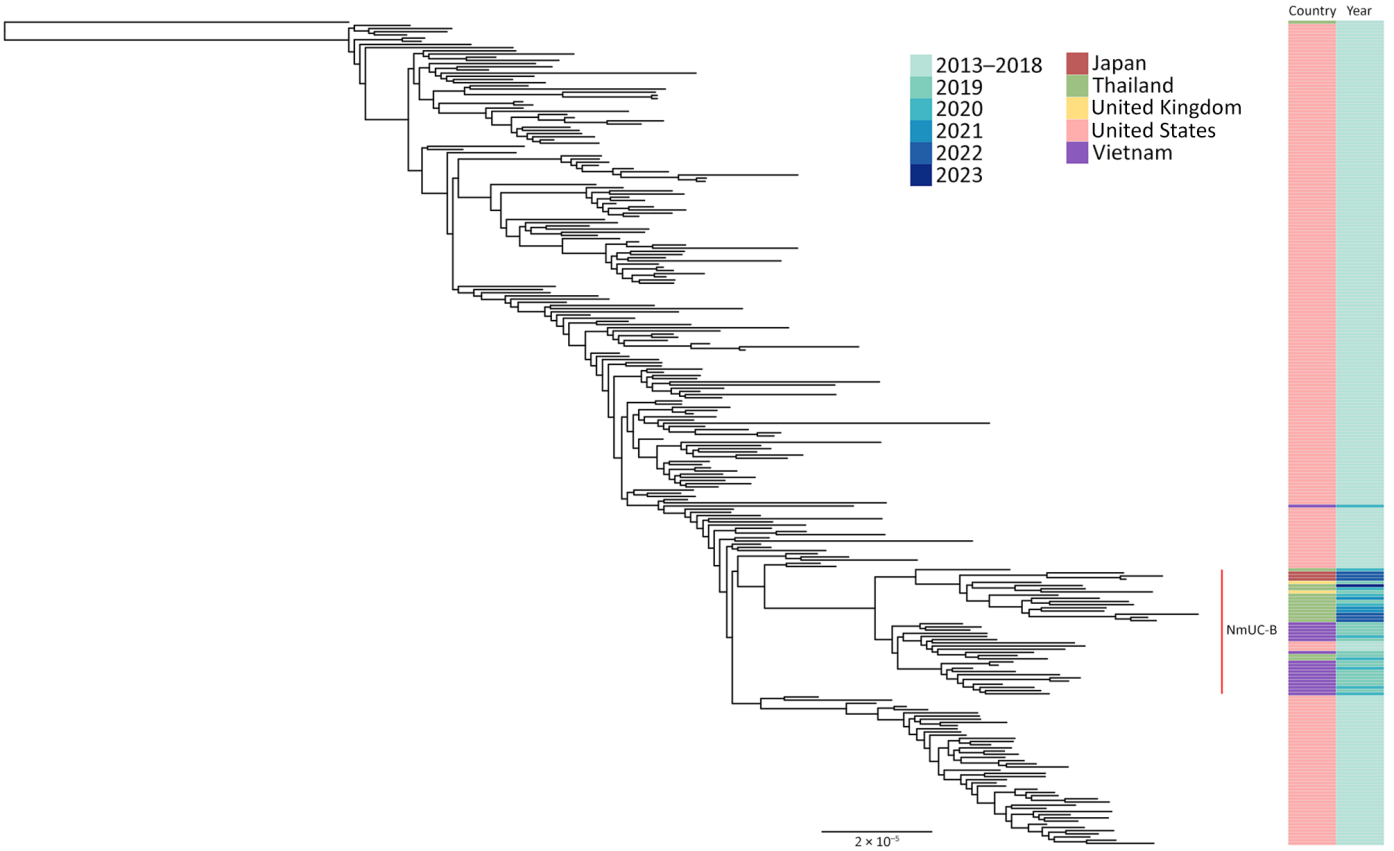
Maximum-likelihood phylogenetic tree from investigation of antimicrobial-resistant clonal complex 11 *Neisseria meningitidis*–associated urethritis cluster, Thailand. The tree is based on core-genome single-nucleotide polymorphisms of 259 clonal complex 11 *N. meningitidis* isolates collected from symptomatic male urethritis patients, representing the *N. meningitidis* urethritis clade. Scale bar indicates nucleotide substitutions per site. NmUC-B, *N. meningitidis* urethritis clade–subclade B.

To clarify the global placement of the CC11 urethral *N. meningitidis* isolates from Thailand, we performed core-genome SNP phylogenetic analysis on the 15 CC11 isolates from Thailand and 241 previously reported global *Nm*UC isolates (Figure 1). Most (14/15) isolates from Thailand formed a monophyletic clade with the newly emerging *Nm*UC-B subclade (*6*). *Nm*UC-B is diverging from the original US *Nm*UC, and isolates from *Nm*UC-B were collected in Europe and Asia during 2019–2023. Of note, we found the first *N. meningitidis* isolate (NM1) collected from Thailand’s EGASP activities in 2017 formed an outgroup for the entire *Nm*UC CC11 and is distantly related to the rest of the isolates from Thailand that clustered in the *Nm*UC-B subclade.

**Figure 2:**
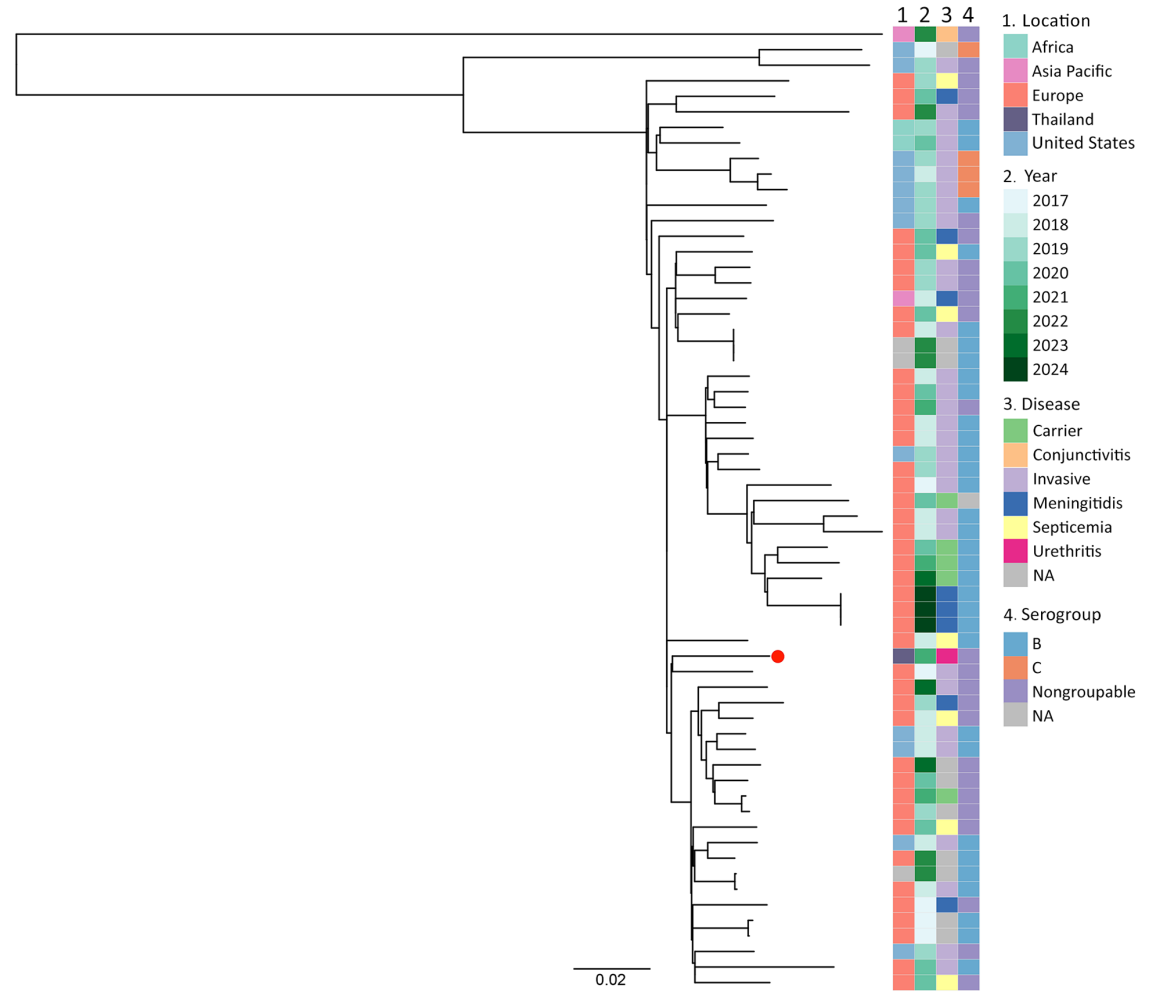
Maximum-likelihood phylogenetic tree of clonal complex 35 *Neisseria meningitidis* isolates used in investigation of antimicrobial-resistant clonal complex 11 *N. meningitidis*–associated urethritis cluster, Thailand. The tree is based on 65 core-genome single-nucleotide polymorphisms of isolates collected from multiple disease types. Red dot indicates the single *N. meningitidis*–associated urethritis isolate (NM4) from this study. Scale bar indicates nucleotide substitutions per site. NA, not available.

We genotyped 1 isolate (NM4) as CC35 and found it was distantly related to the CC11 isolates. We conducted a phylogenetic analysis of that CC35 isolate and 63 CC35 isolates collected during 2017–2024 from Africa, Asia Pacific, Europe, and the United States (Figure 2). The other 63 isolates were collected from multiple disease types, including conjunctivitis, invasive infections, meningitidis, and septicemia, and from asymptomatic carriers. Isolate NM4 was the only isolate from a urethritis case and was most closely related (141 SNPs difference) to an isolate collected in Germany in 2017 from an invasive infection case.

We conducted antimicrobial susceptibility testing for azithromycin, cefixime, ceftriaxone, ciprofloxacin, and gentamicin on the 16 selected isolates by using Etest Antibiotic Susceptibility Testing Reagent Strips (bioMérieux, https://www.biomerieux.com). We determined categorical MICs for *N. meningitidis* by following published guidelines (*13*). Most (15/16) isolates were susceptible to all antimicrobial drugs (Table). Three isolates from the *Nm*UC-B subclade had an elevated MIC or resistance to ciprofloxacin: MIC for isolate NM8 was 0.125 µg/mL, for NM10 was 1.5 µg/mL, and for NM16 was 0.38 µg/mL. Upon further genomic analysis into AMR marker determinants, NM10 carried dual mutations, T91F and D95A, in the *gyrA* allele, and NM16 carried the *gyrA* T91I mutation. Those *gryA* variants have been shown to reduce susceptibility to ciprofloxacin in both *N. gonorrhoeae* and *N. meningitidis* (*5*,*9*).

**Table:** MICs of 16 isolates from investigation of antimicrobial-resistant clonal complex 11 *Neisseria meningitidis*–associated urethritis cluster, Thailand*

Isolate no.	Azithromycin MIC	Cefixime MIC	Ceftriaxone†			Ciprofloxacin‡		Gentamicin MIC
			MIC	CLSI category		MIC	CLSI category	
NM1	0.75	<0.016	<0.002	Susceptible		0.002	Susceptible	1.5
NM2	1	<0.016	0.002	Susceptible		0.003	Susceptible	3
NM3	0.75	<0.016	<0.002	Susceptible		0.003	Susceptible	3
NM4	0.5	<0.016	<0.002	Susceptible		0.002	Susceptible	3
NM5	0.75	<0.016	<0.002	Susceptible		0.003	Susceptible	3
NM6	1	0.016	0.003	Susceptible		0.004	Susceptible	2
NM7	0.25	0.75	0.38	NA		0.006	Susceptible	2
NM8	1	<0.016	<0.002	Susceptible		0.125	Resistant	3
NM9	1	<0.016	<0.002	Susceptible		0.006	Susceptible	3
NM10	0.38	<0.016	<0.002	Susceptible		1.5	Resistant	3
NM11	0.38	<0.016	<0.002	Susceptible		0.003	Susceptible	2
NM12	0.75	<0.016	<0.002	Susceptible		0.003	Susceptible	3
NM13	1	<0.016	<0.002	Susceptible		<0.002	Susceptible	3
NM14	0.38	<0.016	<0.002	Susceptible		0.004	Susceptible	2
NM15	0.5	<0.016	<0.002	Susceptible		0.003	Susceptible	3
NM16	0.25	<0.016	0.002	Susceptible		0.38	Resistant	1.5

*MIC values are µg/mL. Antimicrobial susceptibility testing was conducted by gradient strips. Categorical designations are shown for antimicrobial drugs with official clinical breakpoints for *N. meningitidis* based on CLSI guidelines (*13*). CLSI, Clinical and Laboratory Standards Institute (Wayne, Pennsylvania, USA); NA, not available. †*N. meningitidis* is considered susceptible to ceftriaxone when the MIC values are <0.12 µg/mL. CLSI does not define a resistant category for ceftriaxone for *N. meningitidis* because a resistant breakpoint has not yet been established. ‡ *N. meningitidis* is considered resistant to ciprofloxacin when the MIC values are >0.12 µg/mL.

Isolate NM7, which had elevated MICs to both ceftriaxone and cefixime, harbored the *penA*-2840 allele type. That allele is closely related (93.6% sequence similarity) to the mosaic *penA*-60 allele, which can cause elevated MICs to ceftriaxone and cefixime in *N. gonorrhoeae* (*14*,*15*). Ceftriaxone is the recommended first-line treatment for *N. gonorrhoeae* and *N. meningitidis* infections. NM7 was also part of the *Nm*UC-B subclade.

We also analyzed genomic features associated with *N. meningitidis* urethral colonization. All 16 isolates were nongroupable, and in silico prediction showed they had lost the *N. meningitidis* capsule. Eleven of the 16 isolates carried the insertion sequence element *IS*1301 in the capsule polysaccharide (*cps*) locus, disrupting capsule biosynthesis (Appendix Table 2). We determined that 14 of the 15 CC11 isolates carried the *aniA*/*norB* denitrification cassette associated with microaerobic and anaerobic growth, as well as urethral colonization (Appendix Table 5). Those 14 isolates were all part of the *Nm*UC-B subclade and also harbored the ≈3-kb gonococcal partial operon NEIS1446–NEIS1442 and the gonococcal *argB* (NEIS1038) alleles. The single CC35 isolate did not contain the hallmark genomic features associated with urethral colonization commonly found in the *Nm*UC. However, we found that isolate had lost its capsule and therefore was nongroupable via in silico prediction.

## Conclusions

Our study indicates that the ongoing, global *Nm*UC clade expanded into Thailand as early as 2017. That clade, which originated from a 2015 outbreak in the United States, has not only continued to spread globally but also continued to evolve to create the *Nm*UC-B subclade of isolates that have increased urethral adaptability because of more homologous recombination events with gonococcal DNA (*6*). That increased urethral adaptability could have contributed to the observed increase in the local prevalence of *N. meningitidis*–associated urethritis in Thailand.

We also identified concerning AMR genomic markers with corresponding elevated MICs to both ciprofloxacin and extended-spectrum cephalosporins in the *Nm*UC-B isolates from Thailand. That finding is a public health concern because ciprofloxacin is used as prophylaxis for invasive *N. meningitidis* infections, and extended-spectrum cephalosporins are used to treat *N. gonorrhoeae*–associated urethritis. One isolate had a mosaic *penA* allele that has been associated with decreased susceptibility to ceftriaxone in *N. meningitidis* and cefixime in *N. gonorrhoeae*, demonstrating that *N. meningitidis* could be a reservoir for AMR variants and contribute to the spread of AMR among *Neisseria* spp. bacteria. Those findings raise concerns for both gonococcal and meningococcal disease control. 

In summary, we found increased urethral adaptability and AMR markers among *N. meningitidis* isolates from Thailand. Continued global surveillance is needed to monitor the spread of urethral *N. meningitidis* and the possibility of further AMR in both *N. meningitidis* and *N. gonorrhoeae*.

Appendix Additional information on an antimicrobial-resistant clonal complex 11 *Neisseria meningitidis*–associated urethritis cluster, Thailand.
